# A case of late-onset and long term of anti-NMDA-receptor encephalitis in a 50-year-old patient with culturally acceptable psychosis and cognitive decline

**DOI:** 10.1192/j.eurpsy.2021.1807

**Published:** 2021-08-13

**Authors:** Y. Palchikova, N. Zalutskaya, N. Gomzyakova, V. Mikhailov, N. Ananyeva

**Affiliations:** 1 Geriatric Psychiatry, V. M. BEKHTEREV NATIONAL RESEARCH MEDICAL CENTER FOR PSYCHIATRY AND NEUROLOGY, St.Petersburg, Russian Federation; 2 Of Epilepsy, V.M. Bekhterev National Medical Research Center for Psychiatry and Neurology, Saint Petersburg, Russian Federation; 3 Radiology, V. M. BEKHTEREV NATIONAL RESEARCH MEDICAL CENTER FOR PSYCHIATRY AND NEUROLOGY, St. Petersburg, Russian Federation

**Keywords:** Anti-N-Methyl-D-Aspartate Receptor Encephalitis, cultural psychiatry, Herpes Simplex, psychotic disorder

## Abstract

**Introduction:**

Anti-NMDA-receptor encephalitis is a severe rare acute form of encephalitis caused by an autoimmune process with the synthesis of autoantibodies to the glutamate receptors. The average age of onser is estimated to be 23-25 years. A typical clinical picture consist of prodromal, psychotic, areactive, hyperkinetic phases, and a phase of gradual regression of symptoms. The disease usually lasts for a several weeks with spontaneous recovery or fatal outcome and caused by neoplastic process. Our case demonstrates that the course of anti-NMDAR encephalitis is possible at more mature age in the form of a long process with cultural features, without significant catadrome, inflammation and associated neoplastic process.

**Objectives:**

50-year-old Buddhist Buryat woman with complaints about hypomnesia, anosmia and dissomnia. The disease began with impaired consciousness, disorientation, seizures and memory loss 4 years ago. Then after a visit of Lama, the symtoms described above spontaneously reduced and schizophrenia-like psychosis developed (voice of Lama in the head, a belief in the existence of super-powers, disorganized behavior), mild neurological and severe neurocognitive symptoms, weight loss, intolerance to antipsychotics in minimal daily doses.

**Methods:**

Examination included: CBC, metabolic panel, coagulogram, tumor markers, CSF, MRI, PET, specialists.

**Results:**

CBC, metabolic blood analysis, tumor markers - within the reference values. CSF: cytosis 9/3, glucose 5.5 mmol/l, Pandi++, Nonnet-Apeltau+, antibodies to the NMDA receptor - 8. MRI: signs of the consequences of encephalitis. PET: no signs of metabolic activity of the malignant process.

**Conclusions:**

The diagnosis of anti-NMDA-receptor-encephalitis was confirmed by the laboratory and clinical criteria despite unususal course and age. The trigger role of herpes is possible, but not verified.

**Disclosure:**

No significant relationships.

